# Supplementary material for: Heightened Epstein-Barr virus immunity and potential cross-reactivities in multiple sclerosis
Source: PLoS Pathog. 2024 Jun 6;20(6):e1012177. doi: 10.1371/journal.ppat.1012177 (PMC11156336; doi:10.1371/journal.ppat.1012177)
Supplement: S1 Table — Disease duration was calculated as the year of sampling minus the year of first reported neurological symptom as indicated in patient journal records. EDSS is the last reported score prior to sampling in patient records. (PDF) [file ppat.1012177.s001.pdf]

Supplementary Table 1. Disease duration and EDSS at sampling of pwMS. Disease duration was calculated as the year of sampling minus the year of first reported neurological symptom as indicated in patient journal records. EDSS is the last reported score prior to sampling in patient records.

Donor	Gender	Age	IM	Disease duration (years)	EDSS
MS1	F	33	No	4	2
MS2	F	23	No	1	1
MS3	F	49	No	14	6
MS4	F	39	No	8	0
MS5	F	37	Yes	14	6,5
MS6	F	45	No	1	6
MS7	M	45	Yes	0	2,5
MS8	F	60	No	11	1
MS9	M	30	Yes	3	1
MS10	F	27	No	1	0
MS11	F	47	Yes	1	1
MS12	F	21	No	5	1
MS13	F	41	No	4	1
MS14	F	27	Yes	2	0
MS15	F	32	No	1	2
MS16	F	53	No	32	6
MS17	F	37	Yes	7	0
MS18	F	23	No	5	3,5
MS19	F	36	No	13	2,5
MS20	F	31	No	8	2,5
MS25	M	48	No	15	1
MS26	F	37	No	11	4,5
MS27	F	36	Yes	8	2
MS28	M	44	No	4	1
MS29	F	38	No	3	4,5
MS30	F	40	No	2	0
MS31	F	30	No	3	1
MS32	F	27	No	8	0
MS33	F	41	No	18	5
MS34	M	25	No	1	0
