# Supplementary material for: Heightened Epstein-Barr virus immunity and potential cross-reactivities in multiple sclerosis
Source: PLoS Pathog. 2024 Jun 6;20(6):e1012177. doi: 10.1371/journal.ppat.1012177 (PMC11156336; doi:10.1371/journal.ppat.1012177)
Supplement: S1 Fig — Antibody responses in individuals were correlated with EDSS, disease duration and age. EDSS is the score reported at the time of sampling, disease duration was calculated as the number of years between first reported neurological symptom and date of sampling, and age is the age of individuals at the time of sampling. Spearman correlation coefficient (r) and significant P values are indicated (* p < 0.05). The lines indicate the linear regression slopes and 95% confidence interval of slopes. (PDF) [file ppat.1012177.s002.pdf]

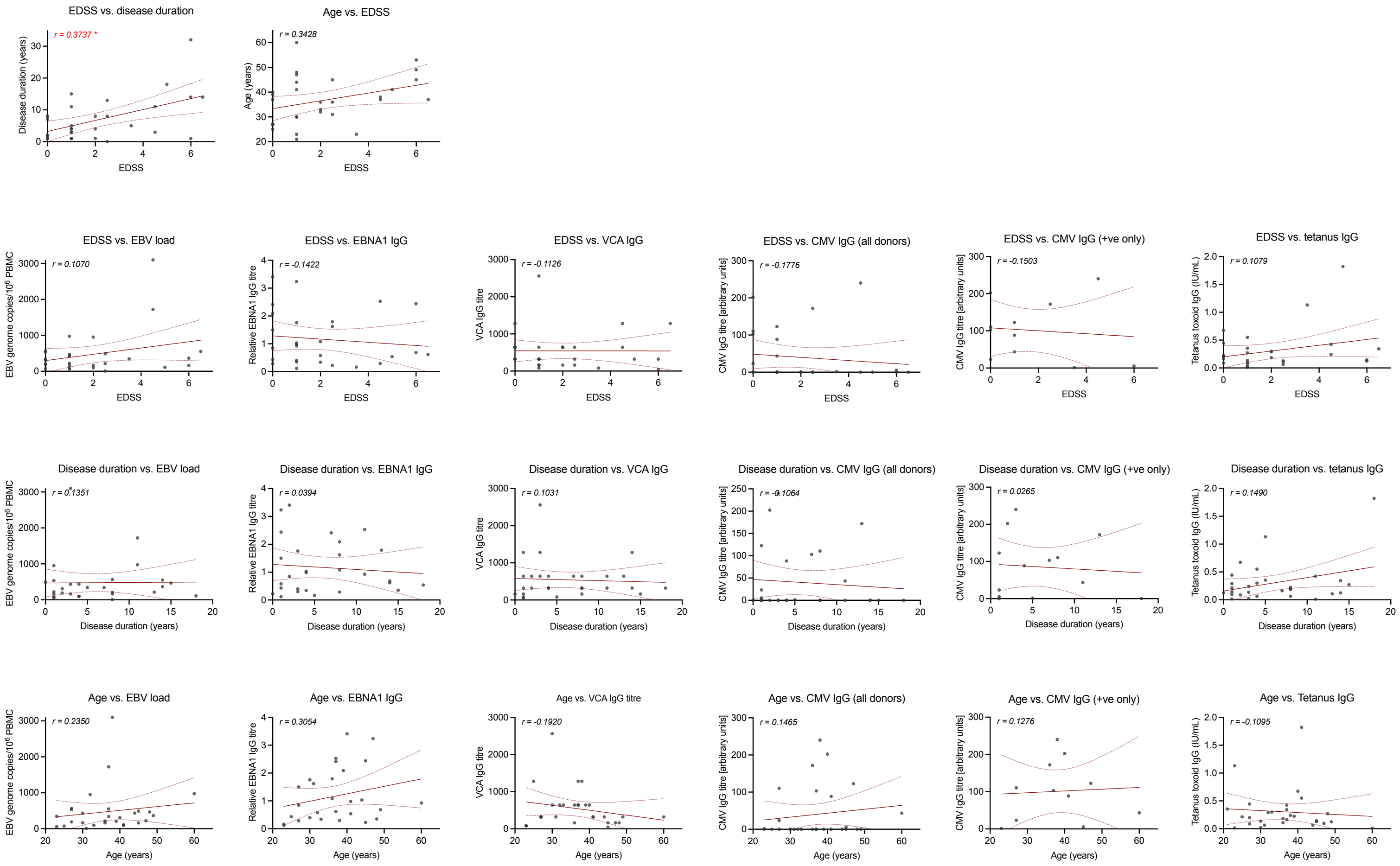

Supplementary Figure 1. Correlation of clinical data with antibody responses in MS. Antibody responses in individuals were correlated with EDSS, disease duration and age. EDSS is the score reported at the time of sampling, disease duration was calculated as the number of years between first reported neurological symptom and date of sampling, and age is the age of individuals at the time of sampling. Spearman correlation coefficient (r) and significant P values are indicated (* $p < 0.05$). The lines indicate the linear regression slopes and 95% confidence interval of slopes.
