# Supplementary material for: Heightened Epstein-Barr virus immunity and potential cross-reactivities in multiple sclerosis
Source: PLoS Pathog. 2024 Jun 6;20(6):e1012177. doi: 10.1371/journal.ppat.1012177 (PMC11156336; doi:10.1371/journal.ppat.1012177)
Supplement: S2 Fig — Ex vivo responding T cells were defined as single, live lymphocytes that were also CD14-CD19-CD3+ before being further divided into CD4+ and CD8+ subpopulations and analysed for cytokine production of IFNγ, IL-2, IL-17 or GMCSF. Data shown is from ex vivo SEB stimulation of donor MS11 PBMC. (PDF) [file ppat.1012177.s003.pdf]

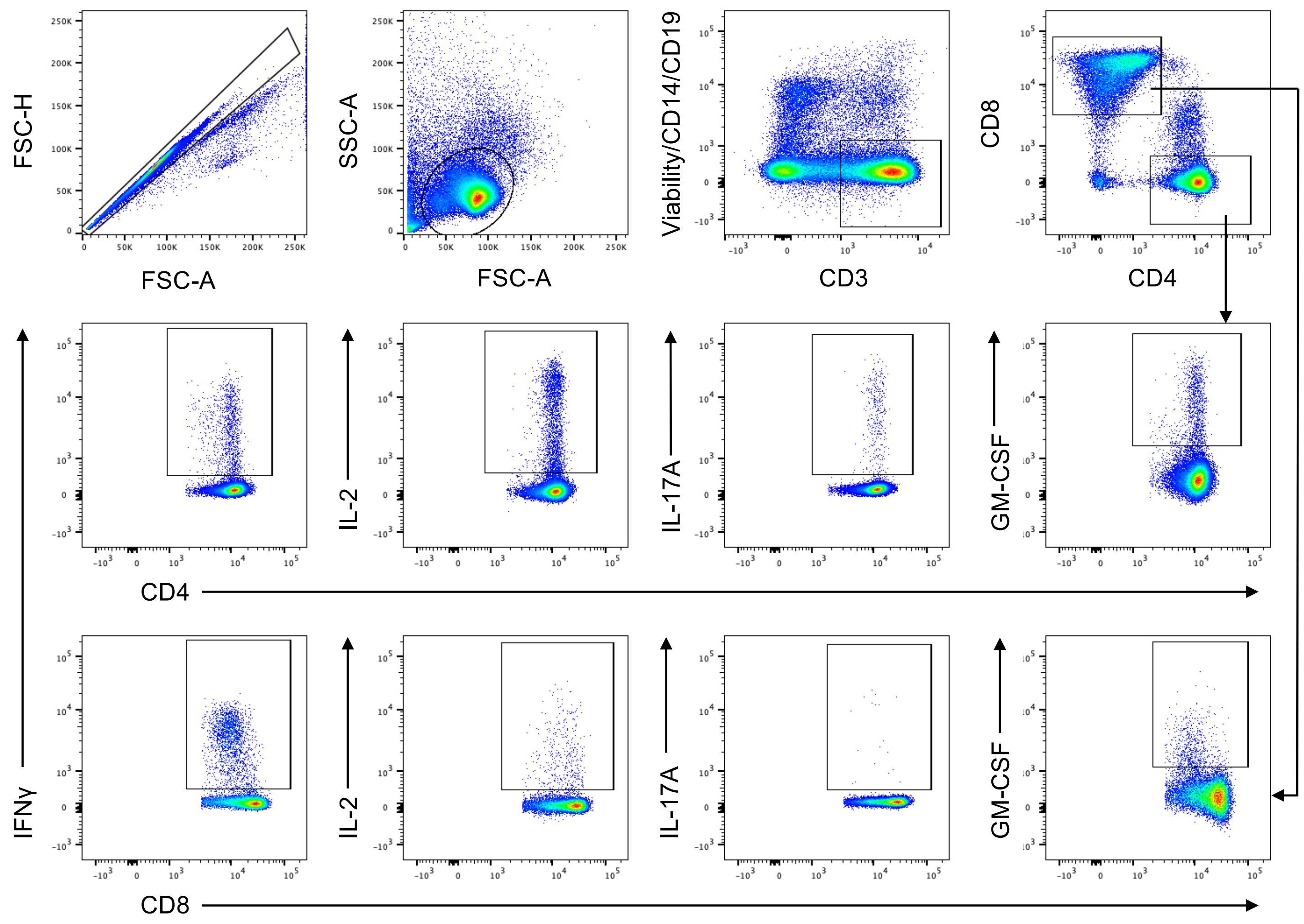

Supplementary Figure 2. Gating strategy for *ex vivo* PBMC to isolate CD4+ and CD8+ T cells responding to different stimuli. *Ex vivo* responding T cells were defined as single, live lymphocytes that were also CD14-CD19-CD3+ before being further divided into CD4+ and CD8+ subpopulations and analysed for cytokine production of IFNγ, IL-2, IL-17 or GM-CSF. Data shown is from *ex vivo* SEB stimulation of donor MS11 PBMC.
