# Supplementary material for: Heightened Epstein-Barr virus immunity and potential cross-reactivities in multiple sclerosis
Source: PLoS Pathog. 2024 Jun 6;20(6):e1012177. doi: 10.1371/journal.ppat.1012177 (PMC11156336; doi:10.1371/journal.ppat.1012177)
Supplement: S3 Fig — Percentage of total CD4+ (A) and CD8+ (B) T cells producing IFNγ, IL-2, IL-17A and GM-CSF in response to ex vivo stimulation with Staphylococcal enterotoxin B (SEB) by ICS (HC n = 20, MS n = 19). Mann-Whitney test, only significant p-values indicated. Ex vivo PBMC were stimulated with autologous WT-LCL, autologous LAT-LCL or EBNA1 peptide pool, and CD4+ and CD8+ T cell responses measured by ICS. Percentage of CD4+ and CD8+ T cells producing IL-17 and GM-CSF in response to EBV antigens is shown in (C-F). Increased IL-17 production from CD8+ T cells after stimulation was seen in post-IM donors compared to HD and MS groups (CD8+IL17+ EBNA1 HD:IM p = 0.0045, MS:IM p = 0.0134). HD n = 27, MS n = 26, post-IM n = 7. Kruskall-Wallis test with Dunn’s multiple comparisons. (* p<0.05, ** p<0.01, *** p<0.001). (PDF) [file ppat.1012177.s004.pdf]

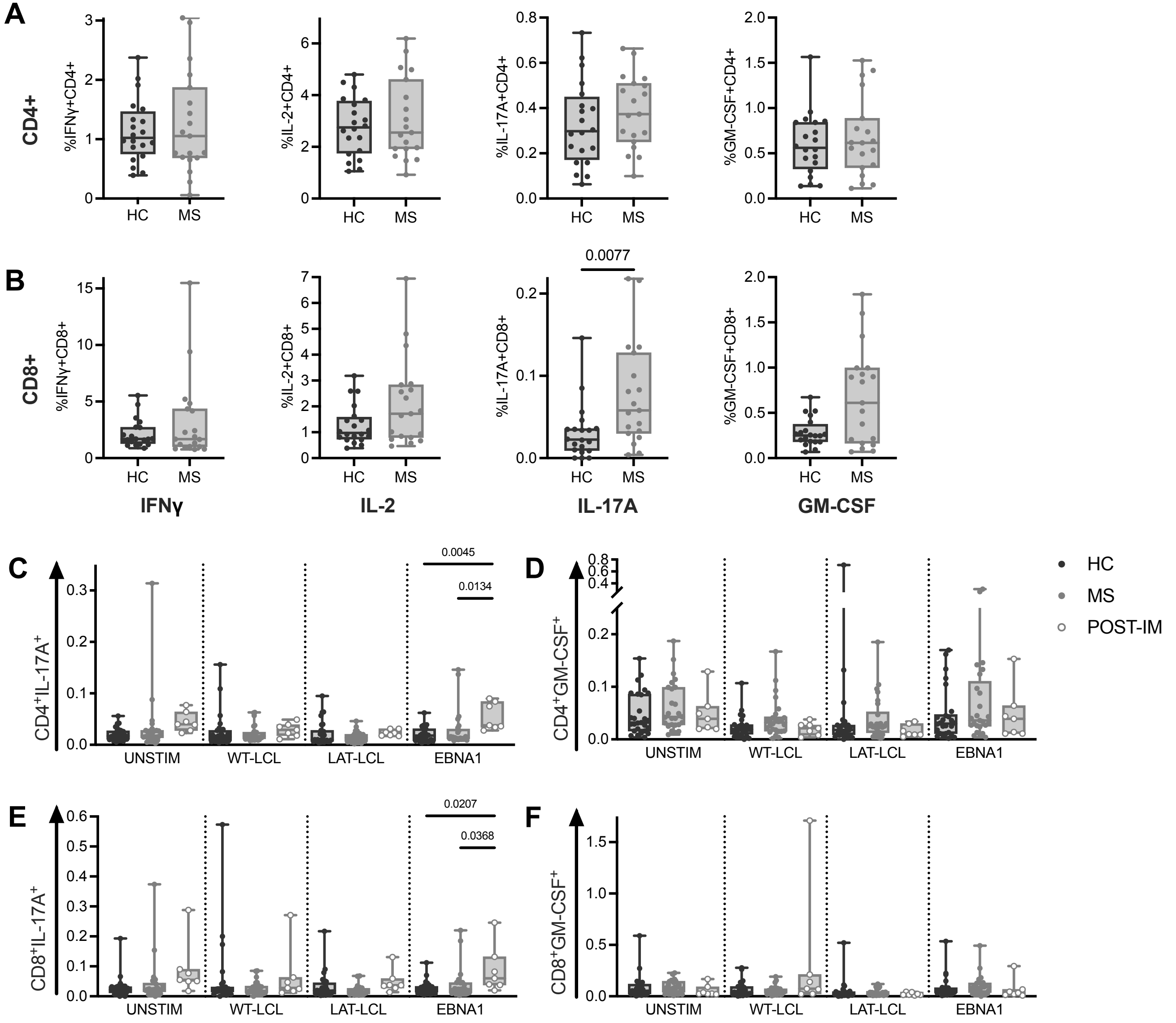

Supplementary Figure 3. Cytokine production in T cells following *ex vivo* stimulation of PBMC with EBV antigens. Percentage of total CD4+ **(A)** and CD8+ **(B)** T cells producing IFNγ, IL-2, IL-17A and GM-CSF in response to *ex vivo* stimulation with Staphylococcal enterotoxin B (SEB) by ICS (HC n=20, MS n=19). Mann-Whitney test, only significant p-values indicated. *Ex vivo* PBMC were stimulated with autologous WT-LCL, autologous LAT-LCL or EBNA1 peptide pool, and CD4+ and CD8+ T cell responses measured by ICS. Percentage of CD4+ and CD8+ T cells producing IL-17 and GM-CSF in response to EBV antigens is shown in **(C-F)**. Increased IL-17 production from CD8+ T cells after stimulation was seen in post-IM donors compared to HD and MS groups (CD8+IL17+ EBNA1 HD:IM *p=0.0045*, MS:IM *p=0.0134*). HD n=27, MS n=26, post-IM n=7. Kruskall-Wallis test with Dunn's multiple comparisons. (* *p<0.05*, ** *p<0.01*, *** *p<0.001*).
