# Supplementary material for: Heightened Epstein-Barr virus immunity and potential cross-reactivities in multiple sclerosis
Source: PLoS Pathog. 2024 Jun 6;20(6):e1012177. doi: 10.1371/journal.ppat.1012177 (PMC11156336; doi:10.1371/journal.ppat.1012177)
Supplement: S4 Fig — Tree plot analysis was performed on T cells responding to SEB, autologous WT-LCL, LAT-LCL or EBNA1 peptide pool and data was analysed using SPICE using permutation and Wilcoxon Signed Rank tests. Analysis of CD4+ T cells shown in (A) and of CD8+ T cells shown in (B). (HD n = 27, MS n = 26, post-IM n = 7, * p<0.05, ** p<0.01, *** p<0.001). (PDF) [file ppat.1012177.s005.pdf]

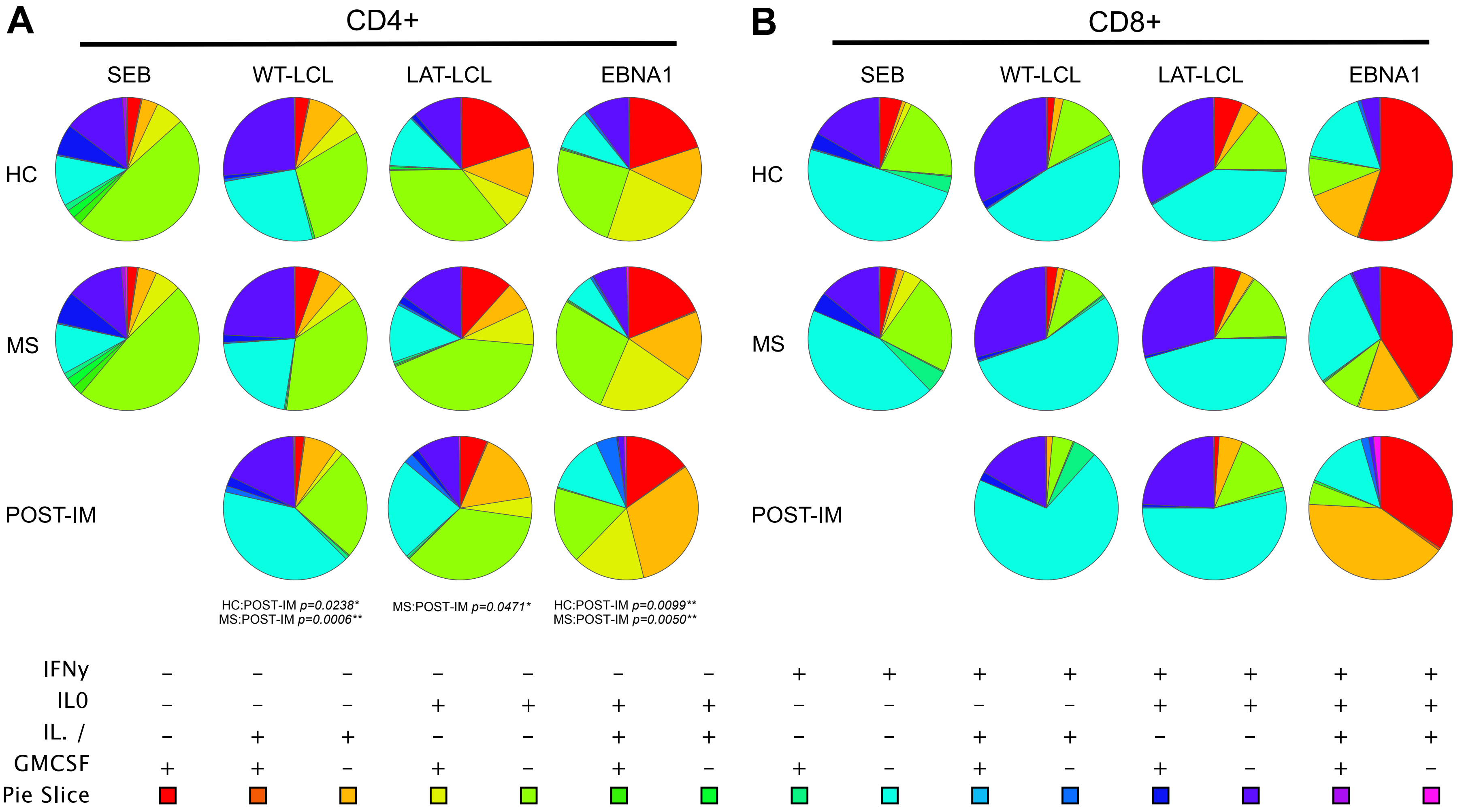

Supplementary Figure 4. Multiple cytokine production of responding CD4+ and CD8+ T cells. Tree plot analysis was performed on T cells responding to SEB, autologous WT-LCL, LAT-LCL or EBNA1 peptide pool and data was analysed using SPICE using permutation and Wilcoxon Signed Rank tests. Analysis of CD4+ T cells shown in **(A)** and of CD8+ T cells shown in **(B)**. (HD n=27, MS n=26, post-IM n=7, * *p<0.05*, ** *p<0.01*, *** *p<0.001).*
