# Supplementary material for: Heightened Epstein-Barr virus immunity and potential cross-reactivities in multiple sclerosis
Source: PLoS Pathog. 2024 Jun 6;20(6):e1012177. doi: 10.1371/journal.ppat.1012177 (PMC11156336; doi:10.1371/journal.ppat.1012177)
Supplement: S5 Fig — Plasma EBNA1 IgG responses were correlated with cytokine production by CD4+ and CD8+ T cells responding to ex vivo EBNA1 peptide pool stimulation from each individual. Plasma EBNA1 IgG was analysed by ELISA and values represent the median of at least 3 separate experiments. (A) IFNγ+CD4+ T cells responding to EBNA1 and EBNA1-specific IgG show a significant correlation of r = 2620 (p = 0.0450). (B) IL-2 cytokine production was not found to be significantly correlated. IFNγ+ (C) and IL-2+ (D) CD8+ T cells responding to EBNA1 and EBNA1-specific IgG were also positively and significantly correlated with r = 0.3437 and r = 0.2901 respectively (IFNγ p = 0.0072, IL-2 p = 0.0245). Spearman’s rank correlation coefficient was calculated (HC n = 26, MS n = 27, IM n = 7) (* p<0.05, ** p<0.01, *** p<0.001). (PDF) [file ppat.1012177.s006.pdf]

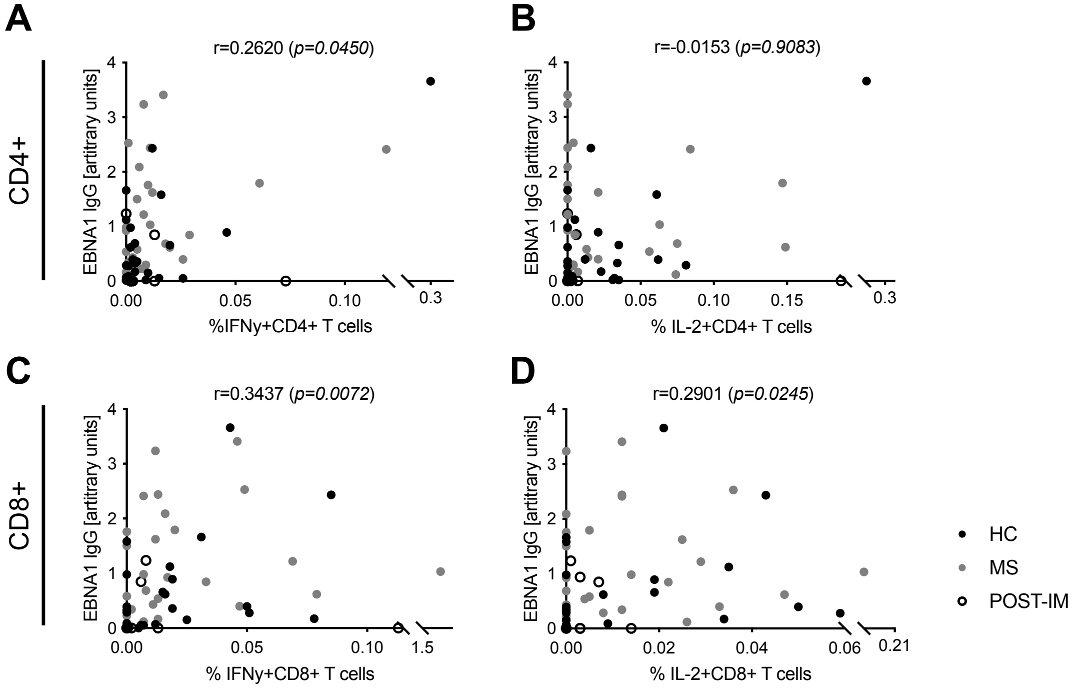


**Supplementary Figure 5. EBNA1-specific T cell and antibody responses are modestly correlated in individuals and all groups.** Plasma EBNA1 IgG responses were correlated with cytokine production by CD4+ and CD8+ T cells responding to *ex vivo* EBNA1 peptide pool stimulation from each individual. Plasma EBNA1 IgG was analysed by ELISA and values represent the median of at least 3 separate experiments. **(A)** IFNγ+CD4+ T cells responding to EBNA1 and EBNA1-specific IgG show a significant correlation of r=2620 (*p=0.0450*). **(B)** IL-2 cytokine production was not found to be significantly correlated. IFNγ+ **(C)** and IL-2+ **(D)** CD8+ T cells responding to EBNA1 and EBNA1-specific IgG were also positively and significantly correlated with r=0.3437 and r=0.2901 respectively (IFNγ *p=0.0072*, IL-2 *p=0.0245*). Spearman's rank correlation coefficient was calculated (HC n=26, MS n=27, IM n=7) (* *p<0.05*, ** *p<0.01*, *** *p<0.001*).
