# Supplementary material for: Heightened Epstein-Barr virus immunity and potential cross-reactivities in multiple sclerosis
Source: PLoS Pathog. 2024 Jun 6;20(6):e1012177. doi: 10.1371/journal.ppat.1012177 (PMC11156336; doi:10.1371/journal.ppat.1012177)
Supplement: S6 Fig — 293 cells were infected with the recombinant MVA viruses expressing CNS antigens, infection was confirmed by expression of green fluorescence protein (GFP) and CNS protein expression was determined by Western blot using an anti-FLAG tag antibody. (A) Absence of GFP+ plaques in uninfected BHK21 cells and GFP+ plaques in BHK21 cells infected with MVA viruses engineered to express recombinant autoantigens. (B) Western blots showing CNS antigen expression in 293 cells infected with recombinant MVA viruses. Expression of CNS proteins is tightly controlled by T7 polymerase co-expression and antigens were only produced when cells were co-infected with the T7 MVA virus. MOBPv1 was unproductive and the construct was made again; MOBPv2 expressed a band of the expected size. Red arrows indicate CNS protein expression of the expected size. (PDF) [file ppat.1012177.s007.pdf]

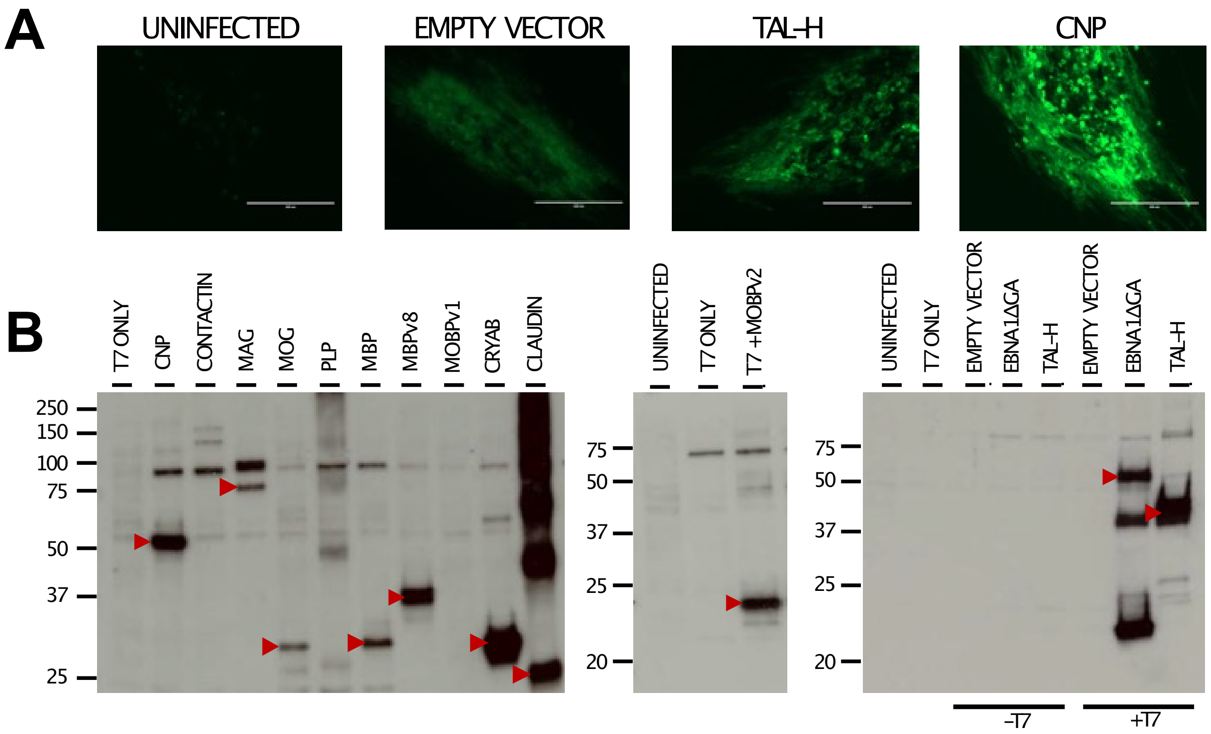

Supplementary Figure 6. Expression of recombinant CNS proteins in 293 cells using the MVA virus system. 293 cells were infected with the recombinant MVA viruses expressing CNS antigens, infection was confirmed by expression of green fluorescence protein (GFP) and CNS protein expression was determined by Western blot using an anti-FLAG tag antibody. **(A)** Absence of GFP+ plaques in uninfected BHK21 cells and GFP+ plaques in BHK21 cells infected with MVA viruses engineered to express recombinant autoantigens. **(B)** Western blots showing CNS antigen expression in 293 cells infected with recombinant MVA viruses. Expression of CNS proteins is tightly controlled by T7 polymerase co-expression and antigens were only produced when cells were co-infected with the T7 MVA virus. MOBPv1 was unproductive and the construct was made again; MOBPv2 expressed a band of the expected size. Red arrows indicate CNS protein expression of the expected size.
