# Supplementary material for: Heightened Epstein-Barr virus immunity and potential cross-reactivities in multiple sclerosis
Source: PLoS Pathog. 2024 Jun 6;20(6):e1012177. doi: 10.1371/journal.ppat.1012177 (PMC11156336; doi:10.1371/journal.ppat.1012177)
Supplement: S7 Fig — Heatmap showing individuals’ CD4+ (A) and CD8+ (B) T cell reactivity to MVA viruses expressing individual CNS antigens. Data from Fig 3. Gray fill indicates missing data. (PDF) [file ppat.1012177.s008.pdf]

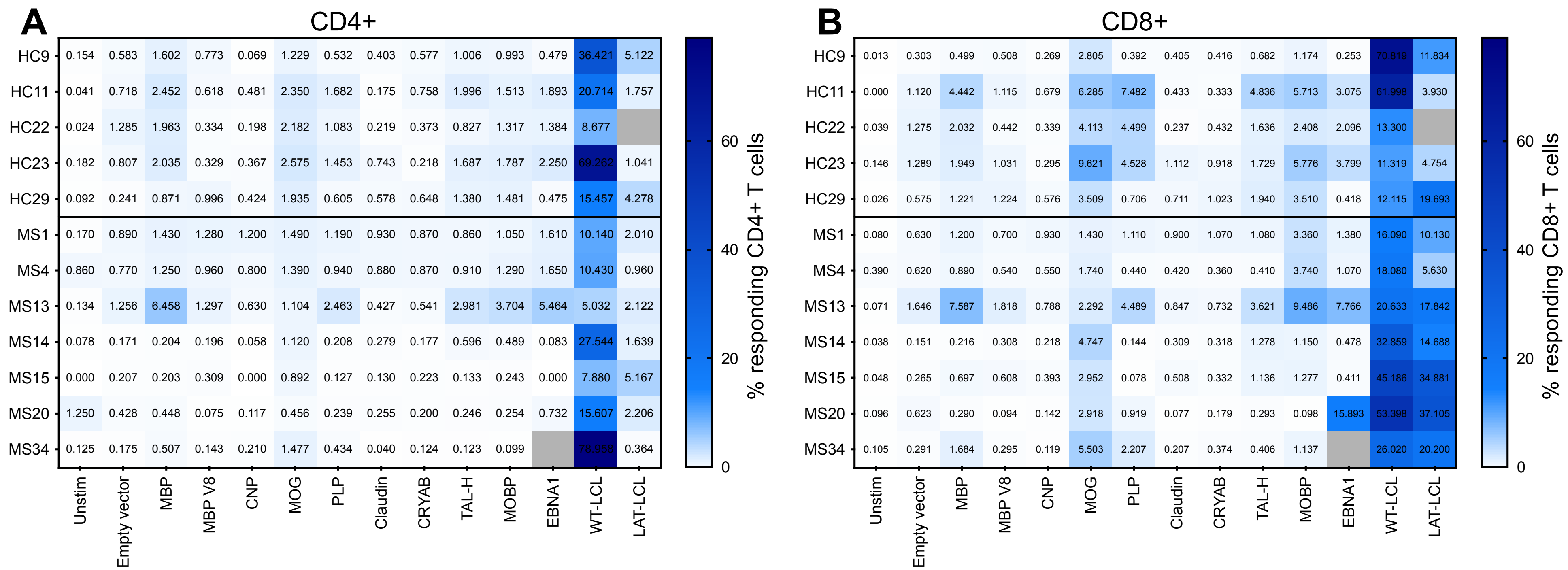

Supplementary Figure 7. Heatmaps showing individuals' WT-LCL-stimulated polyclonal T cell lines reactivity to CNS antigens. Heatmap showing individuals' CD4+ **(A)** and CD8+ **(B)** T cell reactivity to MVA viruses expressing individual CNS antigens. Data from Figure 3. Gray fill indicates missing data.
