# Supplementary material for: Heightened Epstein-Barr virus immunity and potential cross-reactivities in multiple sclerosis
Source: PLoS Pathog. 2024 Jun 6;20(6):e1012177. doi: 10.1371/journal.ppat.1012177 (PMC11156336; doi:10.1371/journal.ppat.1012177)
Supplement: S8 Fig — Whole PBMC were stimulated with autologous wild type LCL on day 0 and day 7. At day 21 LCL-stimulated polyclonal T-cell lines were stimulated with autologous B-cell blasts infected with selected MVAs containing CNS antigens in the presence of TAPI-0. Following stimulation T-cells were surface stained and single, LiveCD19-CD3+TNFα+ cells sorted into 96-well plates. Gating strategy is shown for two representative donors (MS13 and MS14); sorted populations from MOG-stimulated B95.8 polyclonal T-cell lines are shown by the black boxes. (PDF) [file ppat.1012177.s009.pdf]

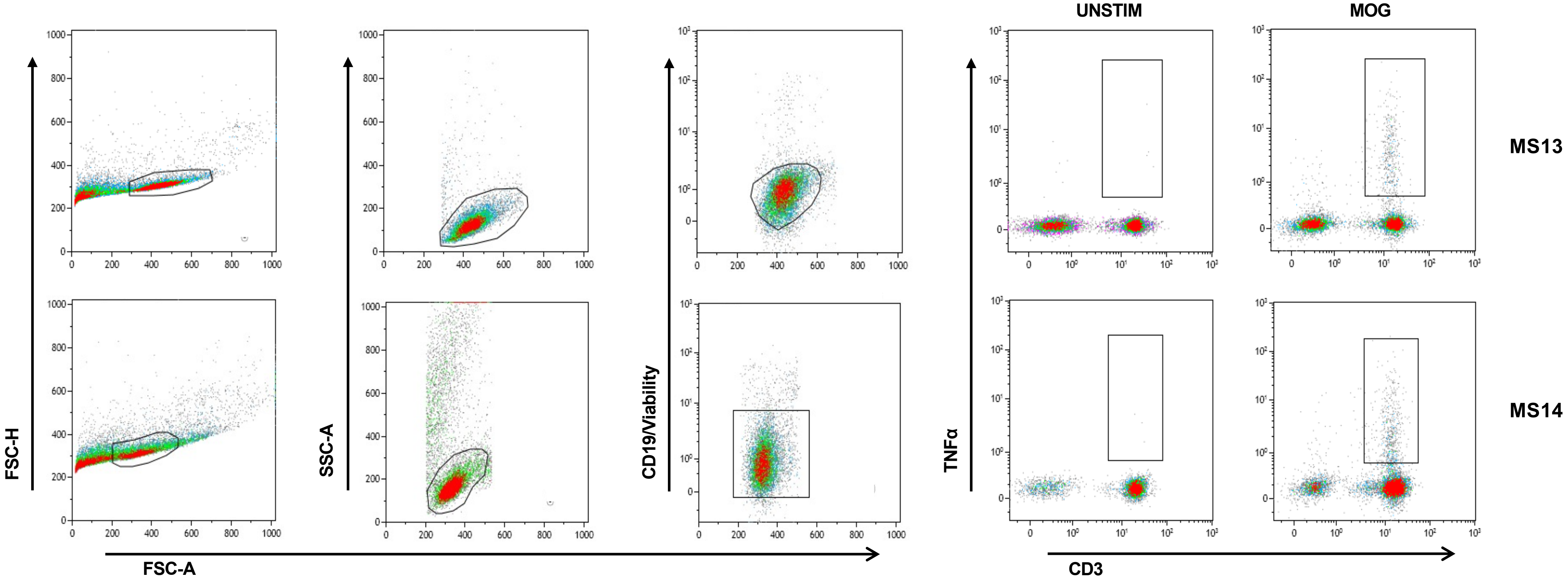

Supplementary Figure 8. Gating strategy for single cell sorting of T-cells with potential dual-specificity for EBV and CNS antigens using TNFα capture. Whole PBMC were stimulated with autologous wild type LCL on day 0 and day 7. At day 21 LCL-stimulated polyclonal T-cell lines were stimulated with autologous B-cell blasts infected with selected MVAs containing CNS antigens in the presence of TAPI-0. Following stimulation T-cells were surface stained and single, LiveCD19-CD3+TNFα+ cells sorted into 96-well plates. Gating strategy is shown for two representative donors (MS13 and MS14); sorted populations from MOG-stimulated B95.8 polyclonal T-cell lines are shown by the black boxes.
