# Supplementary material for: Heightened Epstein-Barr virus immunity and potential cross-reactivities in multiple sclerosis
Source: PLoS Pathog. 2024 Jun 6;20(6):e1012177. doi: 10.1371/journal.ppat.1012177 (PMC11156336; doi:10.1371/journal.ppat.1012177)
Supplement: S9 Fig — T cells expanded in response to autologous WT-LCL were stimulated on day 20 with WT-LCL before flow cytometry and intracellular cytokine staining (ICS). (A) Example IFNγ and TNFα staining of CD4+ and CD8+ T cells from one donor (MS17) WT-LCL-stimulated polylonal T cell line. (B) A high proportion of CD4+ and CD8+ cells from WT-LCL-stimulated polyclonal T cell lines either produced TNFα alone or co-produced IFNγ and TNFα in response to re-stimulation at day 20 with WT-LCL, indicating that we would be likely to capture a high proportion of antigen-specific T cells by using TNFα capture for T cell cloning (n = 5). (C) The proportion of CD4+ and CD8+ T cells producing different combinations of IFNγ and TNFα after re-stimulation with autologous WT-LCL, data produced from 5 WT LCL-stimulated polyclonal T cell lines. (PDF) [file ppat.1012177.s010.pdf]

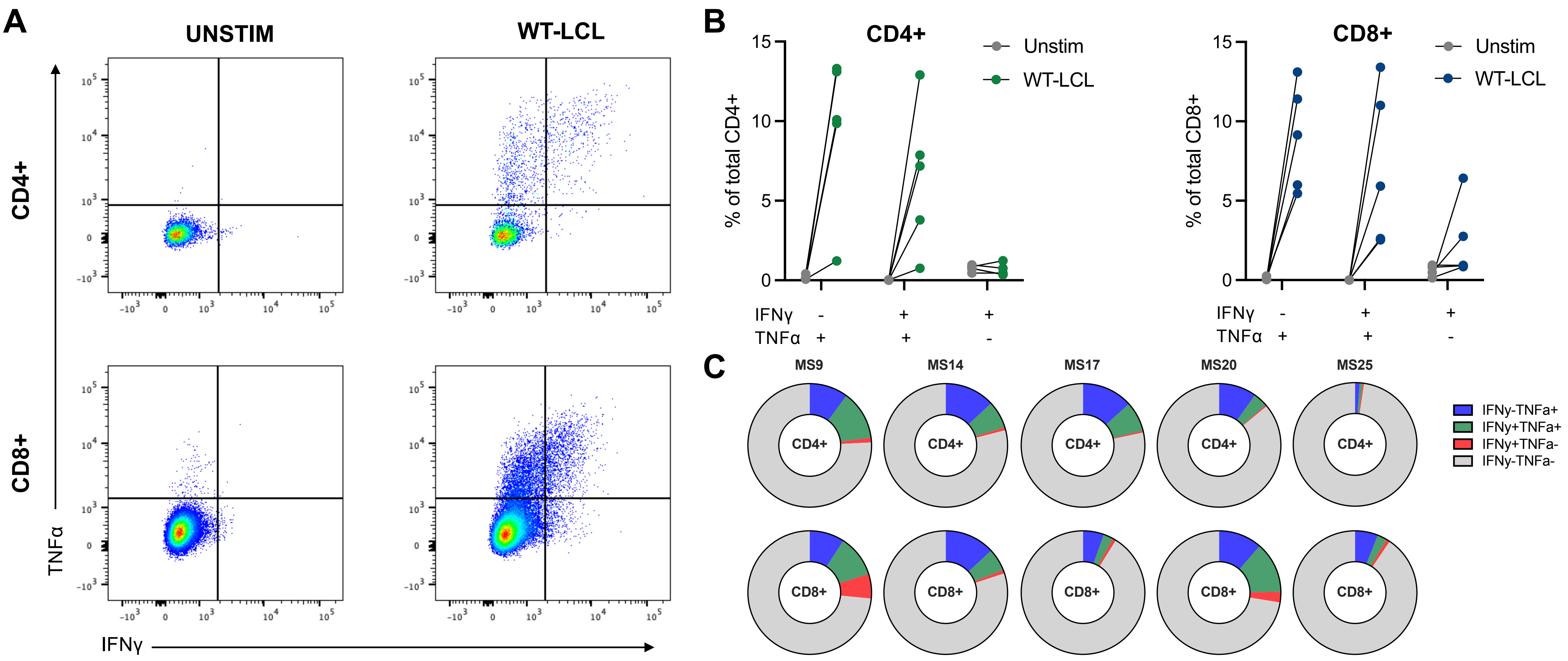

Supplementary Figure 9. Production of IFNγ and TNFα in WT-LCL-specific polyclonal T cell lines after stimulation with WT-LCL. T cells expanded in response to autologous WT-LCL were stimulated on day 20 with WT-LCL before flow cytometry and intracellular cytokine staining (ICS). **(A)** Example IFNγ and TNFα staining of CD4+ and CD8+ T cells from one donor (MS17) WT-LCL-stimulated polylonal T cell line. **(B)** A high proportion of CD4+ and CD8+ cells from WT-LCL-stimulated polyclonal T cell lines either produced TNFα alone or co-produced IFNγ and TNFα in response to re-stimulation at day 20 with WT-LCL, indicating that we would be likely to capture a high proportion of antigen-specific T cells by using TNFα capture for T cell cloning (n=5). **(C)** The proportion of CD4+ and CD8+ T cells producing different combinations of IFNγ and TNFα after re-stimulation with autologous WT-LCL, data produced from 5 WT-LCL-stimulated polyclonal T cell lines.
