# Supplementary material for: Heightened Epstein-Barr virus immunity and potential cross-reactivities in multiple sclerosis
Source: PLoS Pathog. 2024 Jun 6;20(6):e1012177. doi: 10.1371/journal.ppat.1012177 (PMC11156336; doi:10.1371/journal.ppat.1012177)
Supplement: S1 Appendix — (PDF) [file ppat.1012177.s011.pdf]

Supplementary data

Heightened Epstein-Barr virus immunity and potential cross-reactivities in multiple sclerosis

EBNAprint Western blots

Western blots were cropped to remove donor-identifying information, where this was not possible black rectangles indicate redacted information.

EBV seropositive reference plasma (EBVpos CTRL)
EBV seronegative reference plasma (EBVneg CTRL)

Blot 1 – Identification of EBV latent antigen bands using monoclonal antibodies and reference plasma

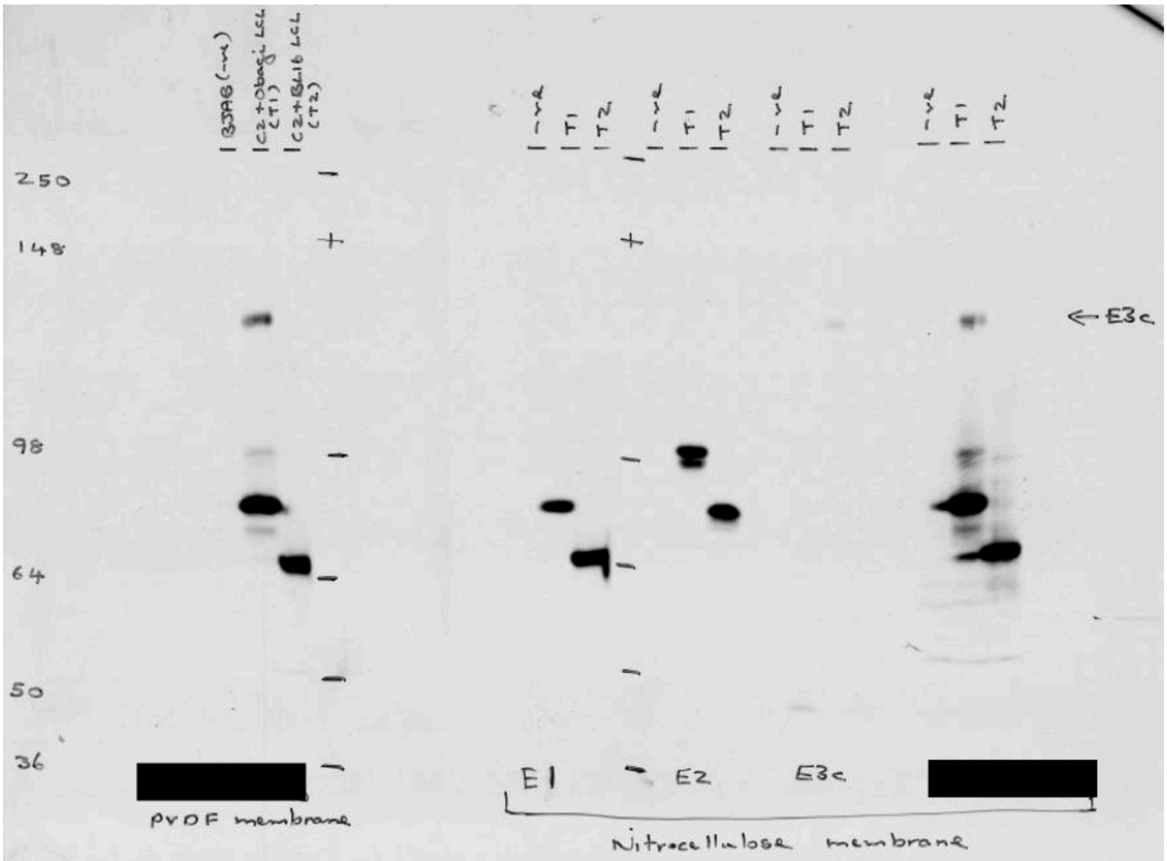

(Left to right) EBVpos CTRL – EBNA1 – EBNA2 – EBNA3C – EBVpos CTRL (repeat)

Blot 2 – Identification of EBV latent antigen bands using monoclonal antibodies and reference plasma

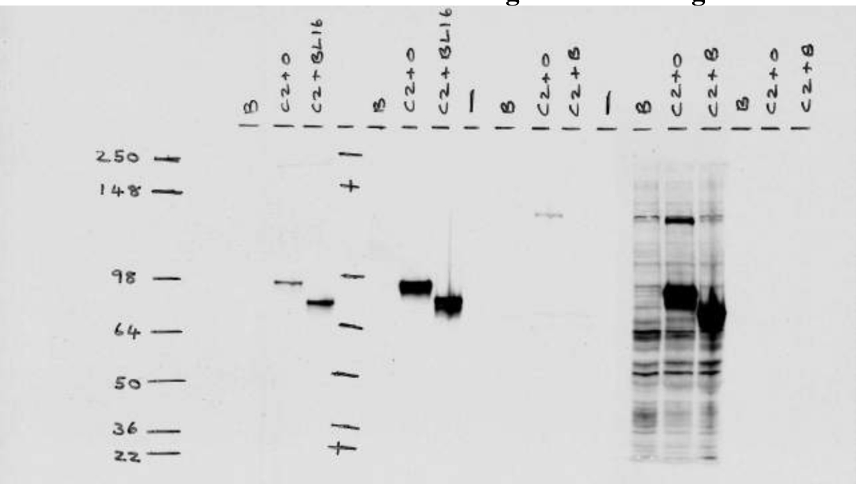

(Left to right): EBNA1 – EBNA2 – EBNA3C – EBVpos CTRL – EBVneg CTRL

Blot 3

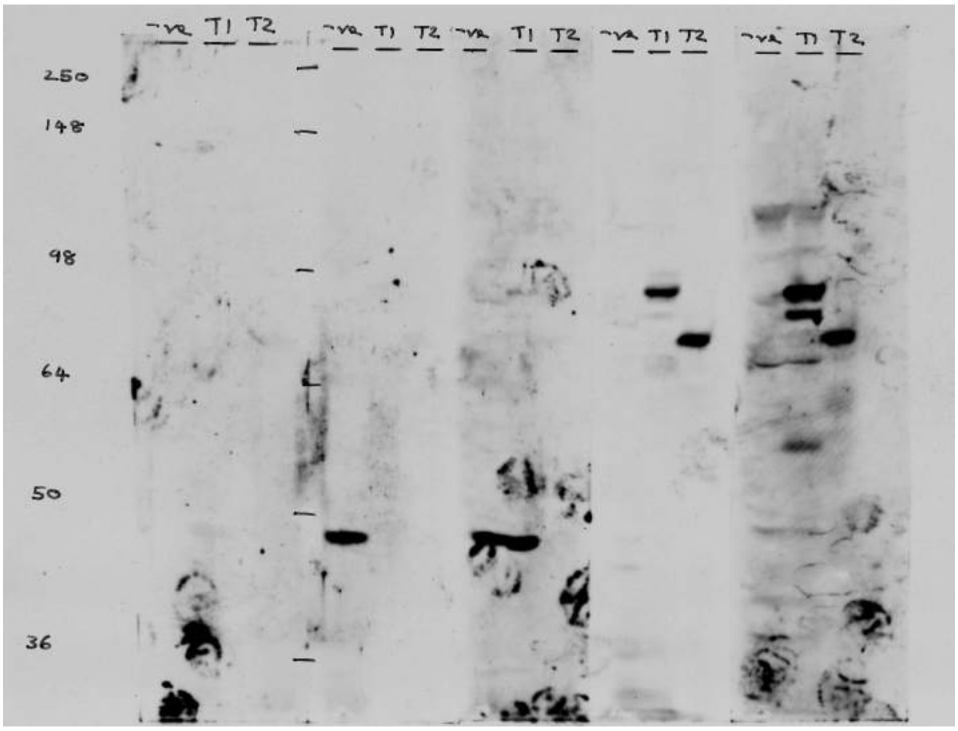

(Left to right) HC7 – HC8 – HC8 (repeat) – MS3 – HC12

Blot 4

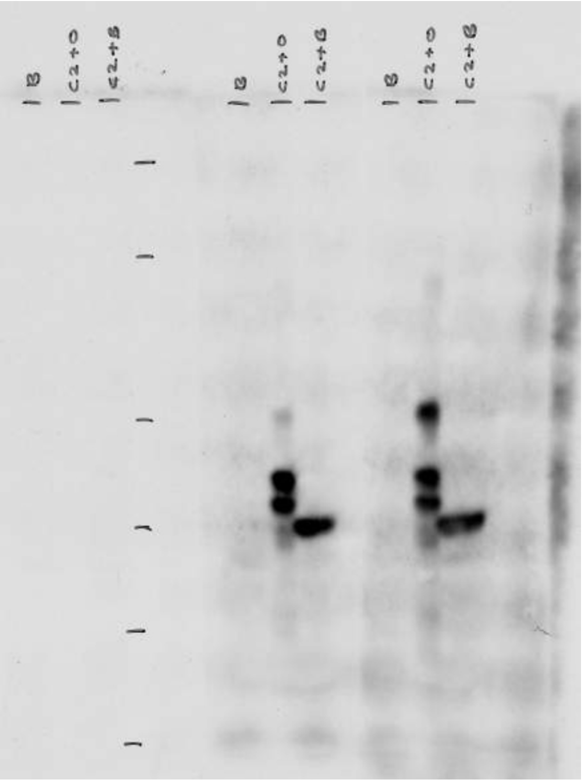

(Left to right) HC7 – HC25 – HC18

Blot 5

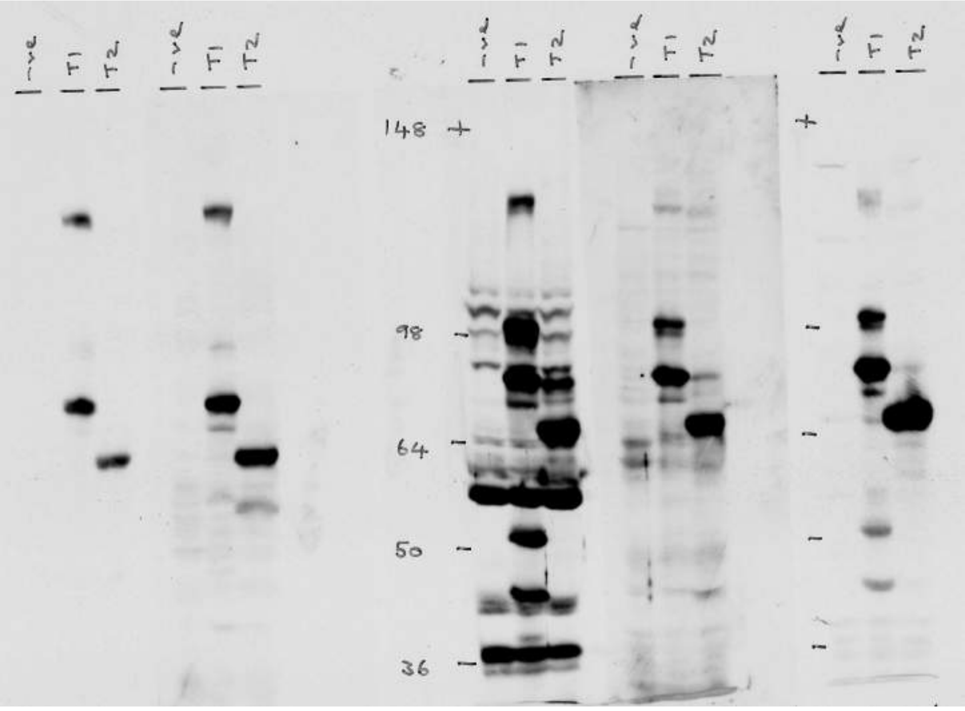

(Left to right) EBVpos CTRL – MS25 – (space) – MS26 – MS27 – MS28

Blot 6

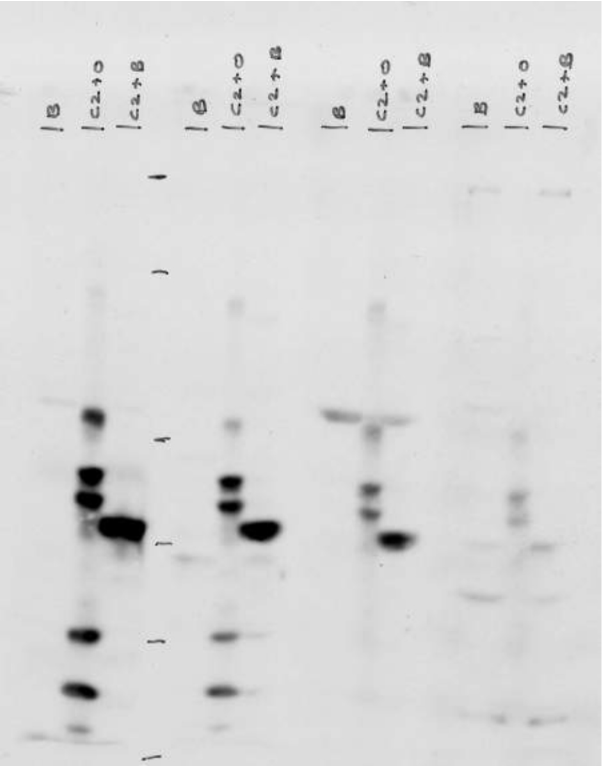

(Left to right) MS29 – MS30 – MS31 – MS32

Blot 7

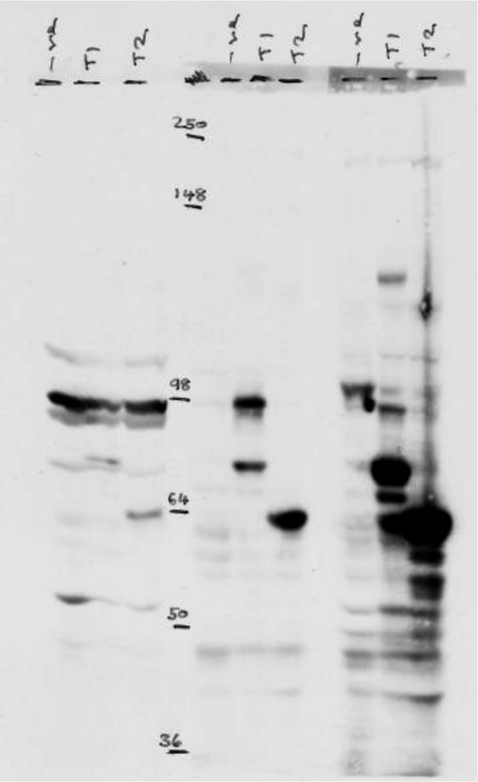

(Left to right) MS2 – MS3 – MS4

Blot 8

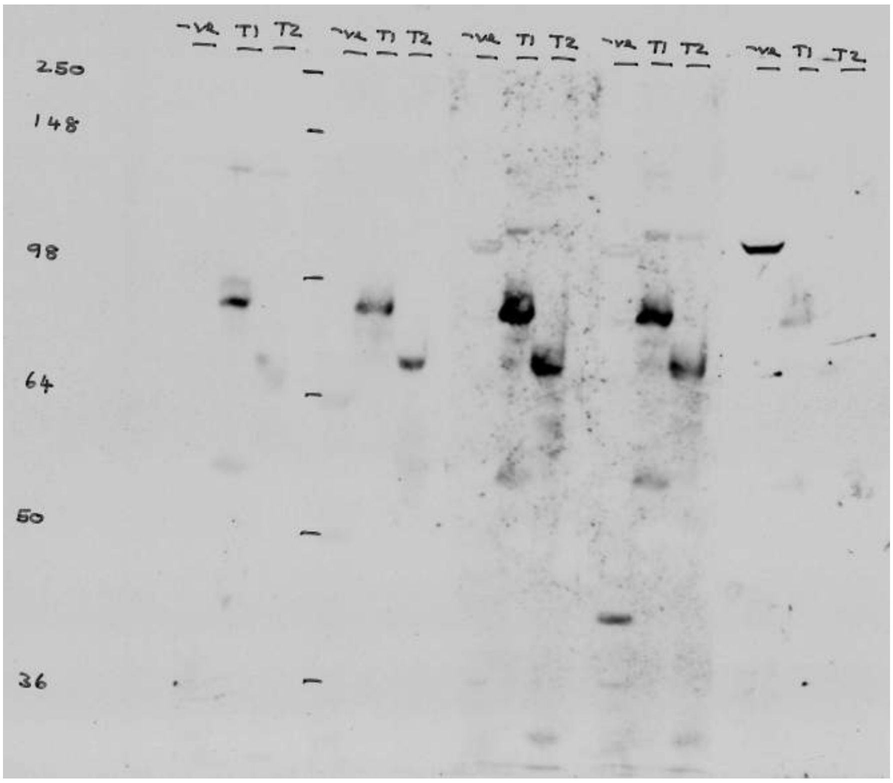

(Left to right) MS5 – MS9 – MS20 – MS20 (repeated) – HC9

Blot 9

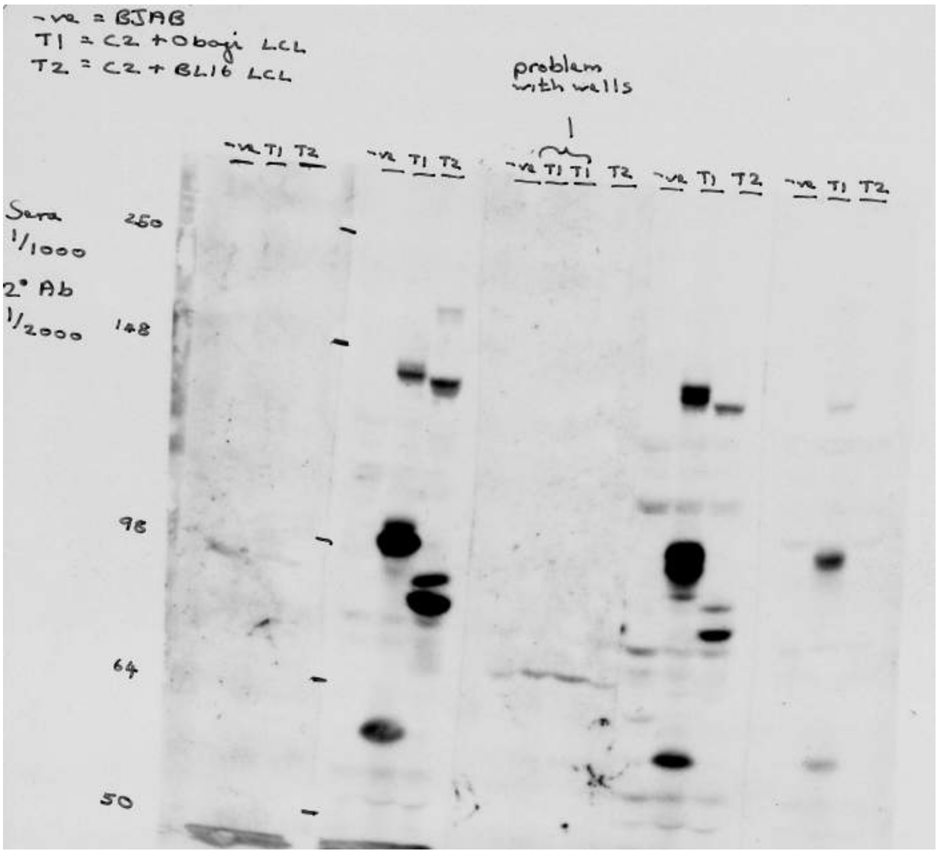

(Left to right) EBVneg CTRL – MS6 – HC7* – IM225 – IM226
*not included due to problem with wells, repeated in other blots

Blot 10

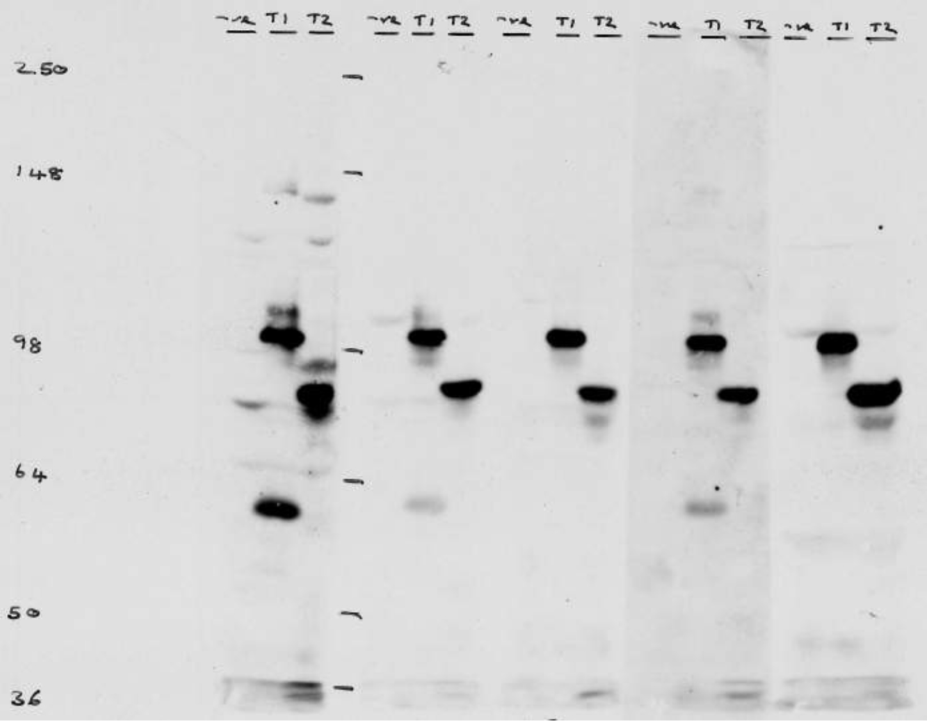

(Left to right) MS6 – MS7 – MS10 – MS13 – MS14

Blot 11

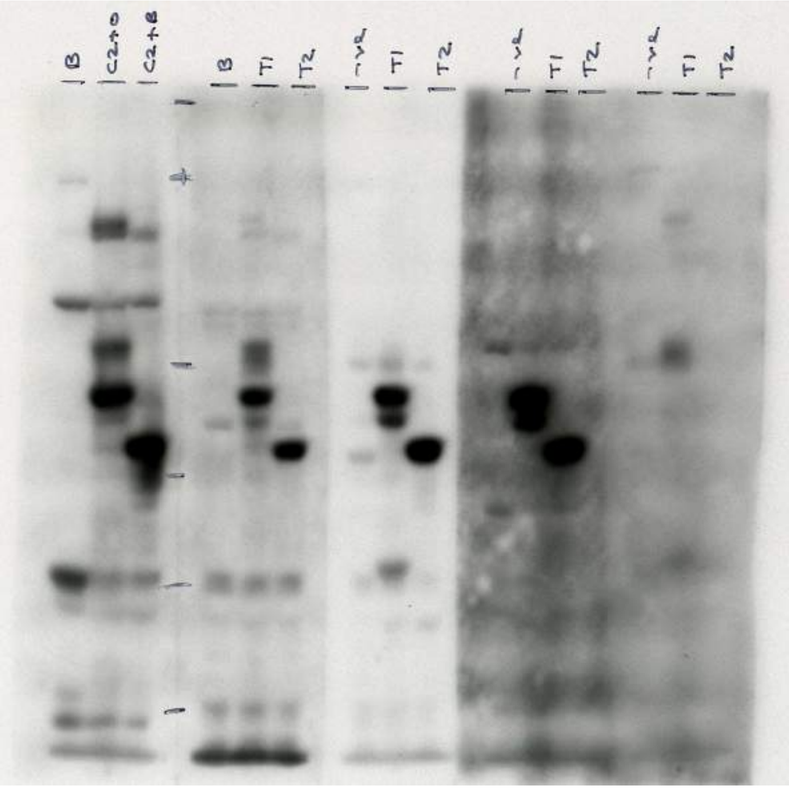

(Left to right) MS15 – MS3 – MS7 – MS10 – HC21

Blot 12

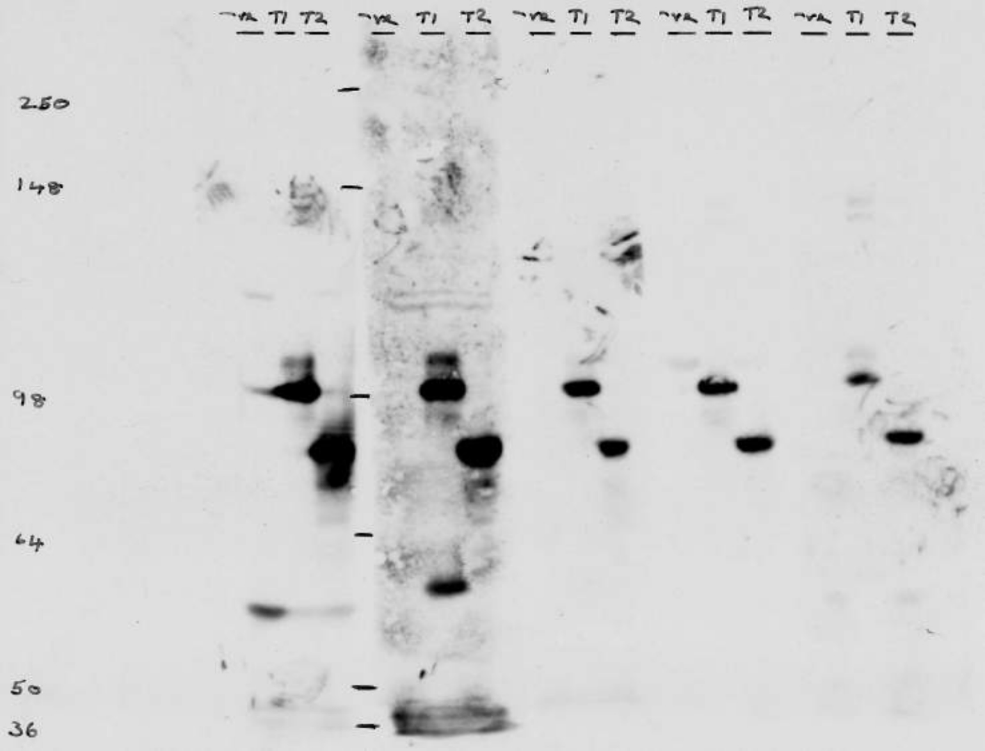

(Left to right) MS15 – MS20 – MS33 – MS34 – MS35

Blot 13

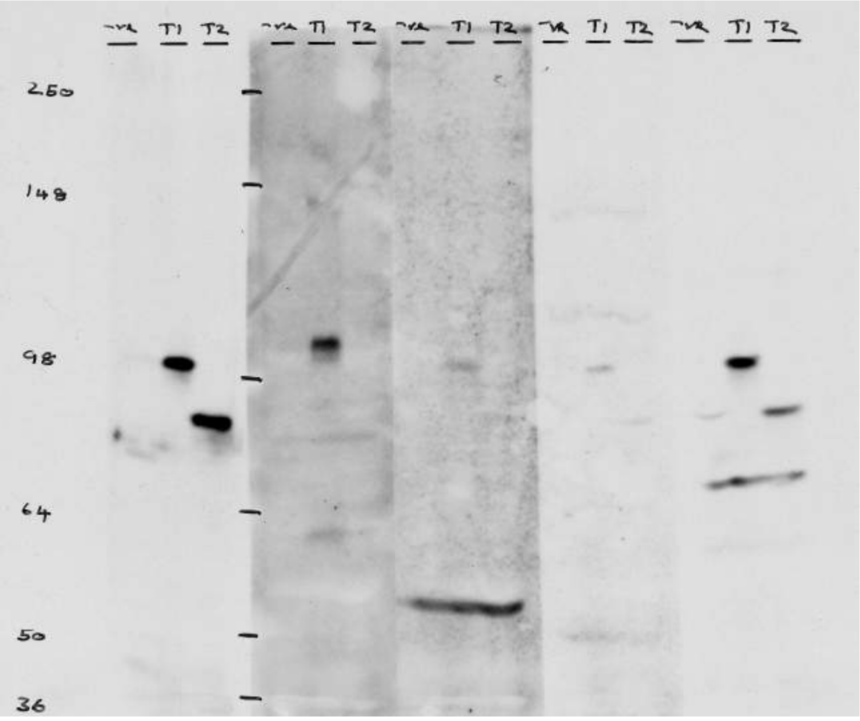

(Left to right) HC1 – HC21 – HC8 – HC22 – HC31

Blot 14

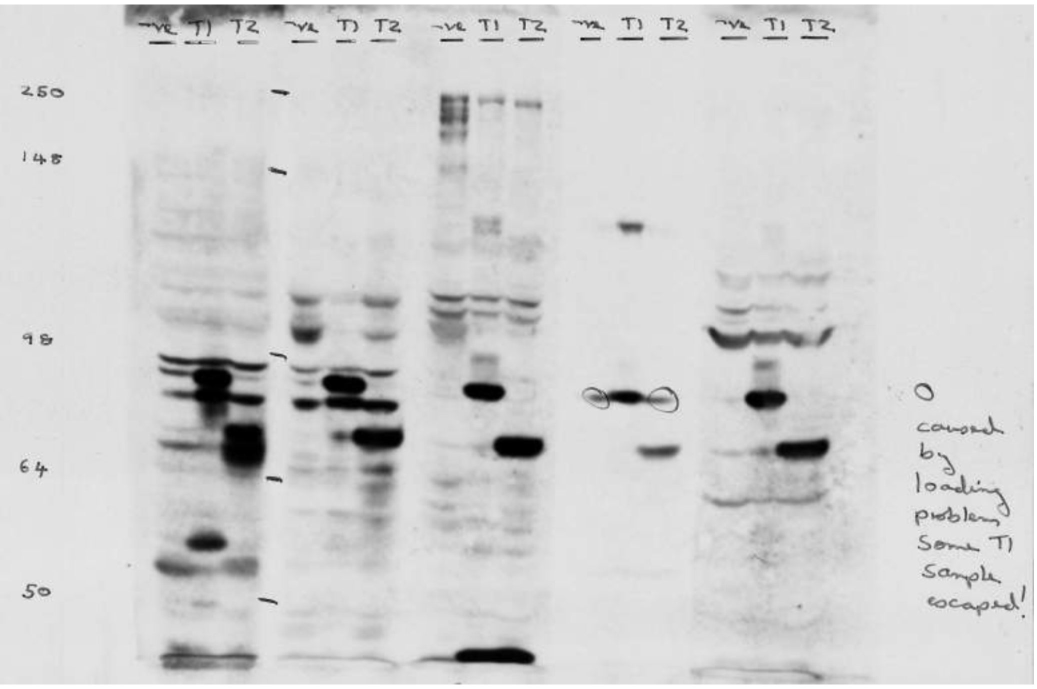

(Left to right) HC4 – HC5 – HC33 – EBVpos CTRL – HC25

Blot 15

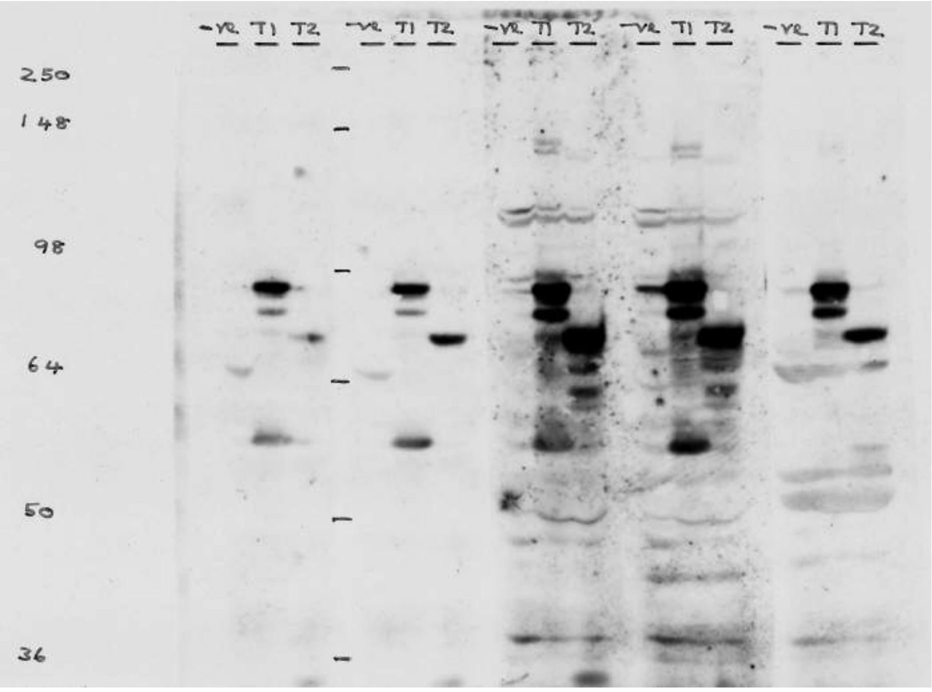

(Left to right) HC11 – HC11 (repeat) – MS20 – MS20 (repeat) – MS9
