# Supplementary material for: Heightened Epstein-Barr virus immunity and potential cross-reactivities in multiple sclerosis
Source: PLoS Pathog. 2024 Jun 6;20(6):e1012177. doi: 10.1371/journal.ppat.1012177 (PMC11156336; doi:10.1371/journal.ppat.1012177)
Supplement: S2 Appendix — (PDF) [file ppat.1012177.s012.pdf]

## Supplementary data

## Heightened Epstein-Barr virus immunity and potential cross-reactivities in multiple sclerosis

## EBNA3 MVA Western blots

Western blots were cropped to remove donor-identifying information. Western blots for some donors were repeated to determine reproducibility or if results were uncertain.

Each donor's plasma was incubated with the following cell lysates in the same order (left to right):

1. BJAB uninfected
2. BJAB MVA EBNA3A-infected
3. BJAB MVA EBNA3B-infected
4. BJAB MVA EBNA3C-infected

**Blot 1**

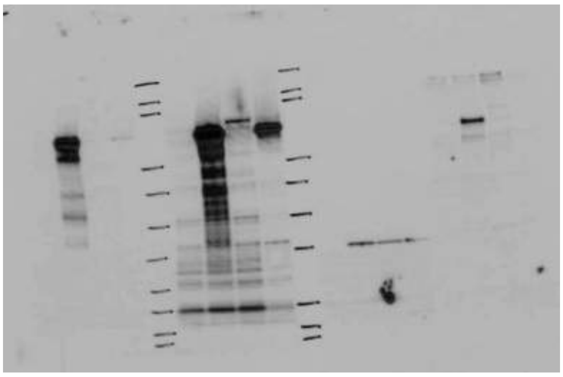

(Left to right) MS17 – MS19 – HC30 – HC33

**Blot 2**

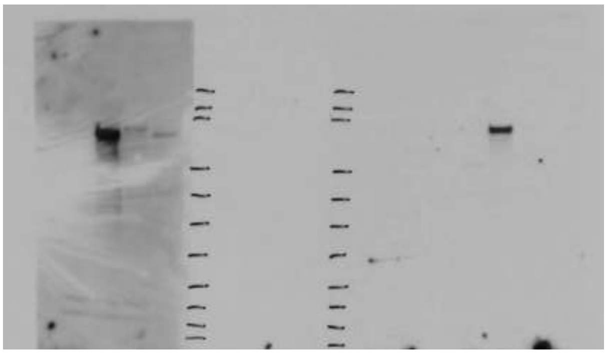

(Left to right) MS35 – empty – HC11 – HC23

**Blot 3**

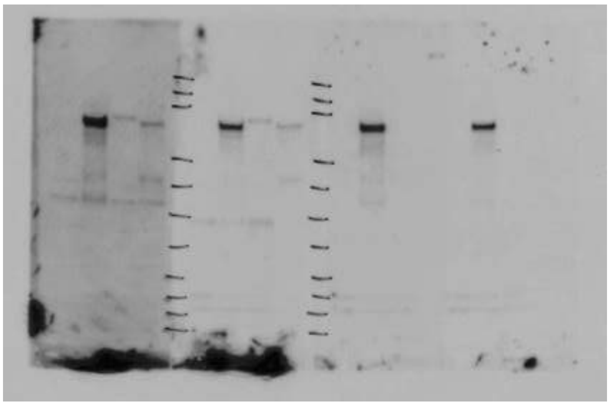

(Left to right) IM235 – HC27 – HC3 – HC3 (repeat)

**Blot 4**

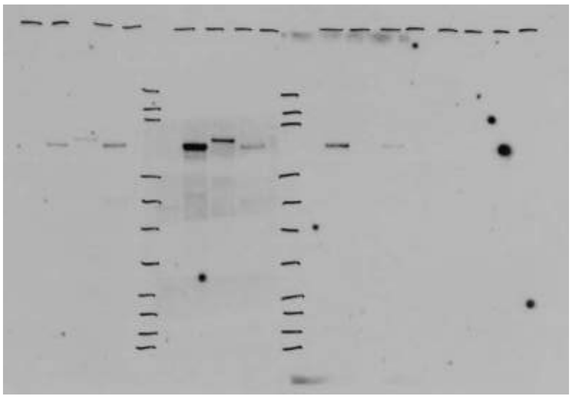

(Right to left) MS4 – HC18 – IM239.3 – HC17

**Blot 5**

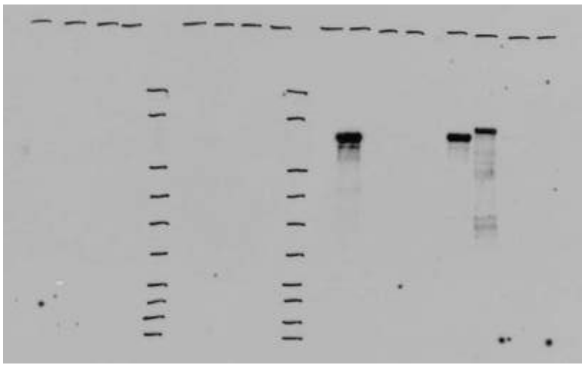

(Right to left) HC15 – IM275.3 – MS6 – MS29

**Blot 6**

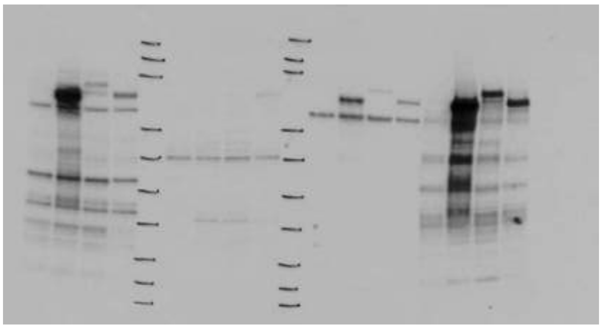

(Left to right) MS15 – HC20 – MS31 – IM225.2

**Blot 7**

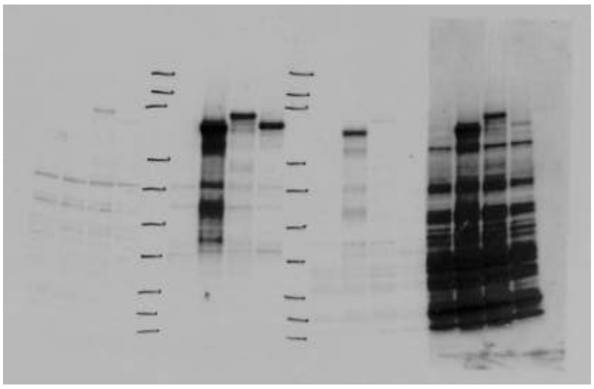

(Left to right) MS9 – IM279.6 – IM226.5 – IM269.3

**Blot 8**

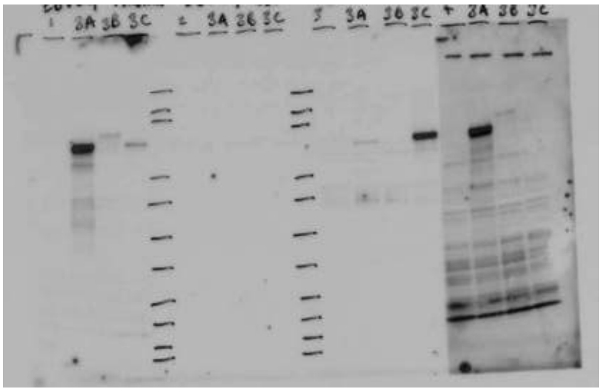

(Left to right) MS35 – empty – HC28 – IM265.7

**Blot 9**

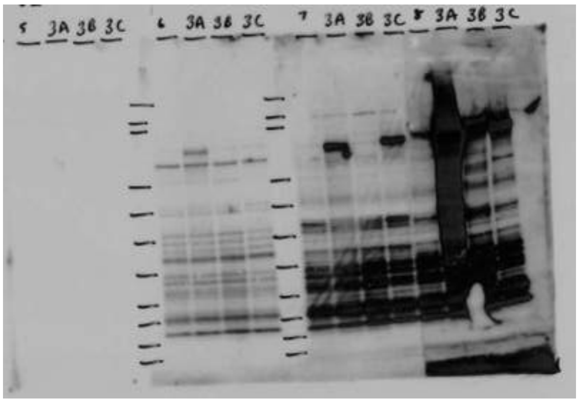

(Left to right) HC11 – IM270.4 – MS8 – MS18

**Blot 10**

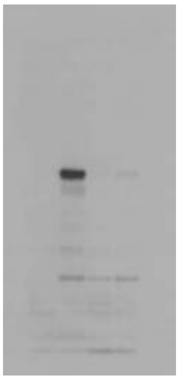

HC6

**Blot 11**

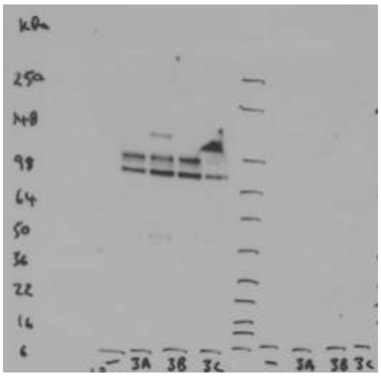


(Left to right) HC5 – HC11

**Blot 12**

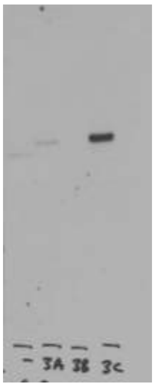


HC28

**Blot 13**

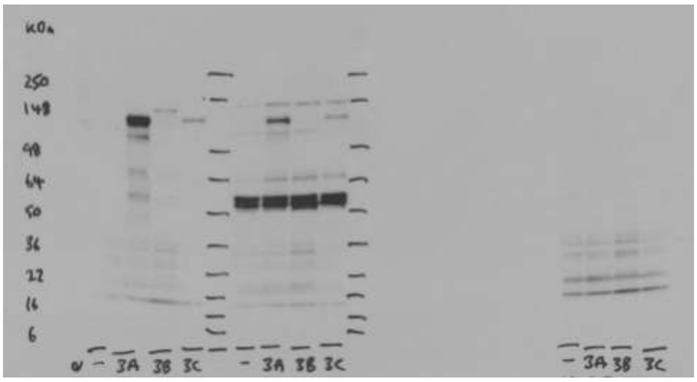


(Left to right) MS35 – HC32 – empty – IM272.2

**Blot 14**

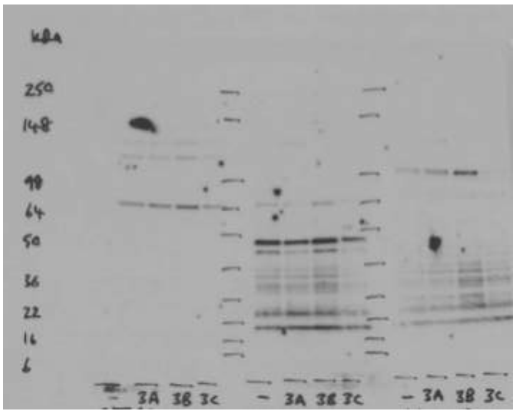


(Left to right) HC32 – IM267.5 – IM238.3

**Blot 15**

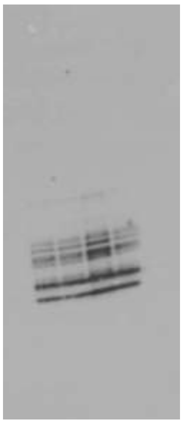

(Left to right) IM257.3

**Blot 16**

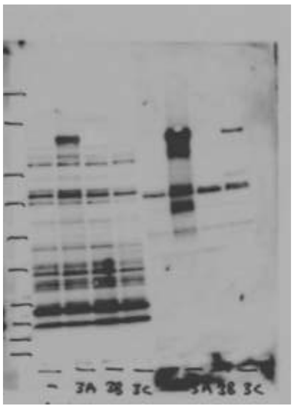


(Left to right) MS10 – HC10

**Blot 17**

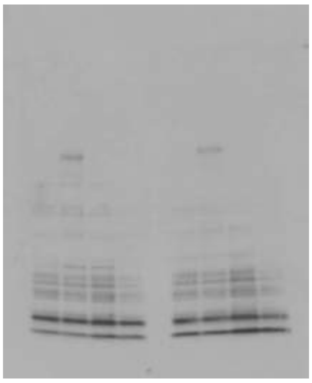

(Left to right) IM243.2 – IM240.2

**Blot 18**

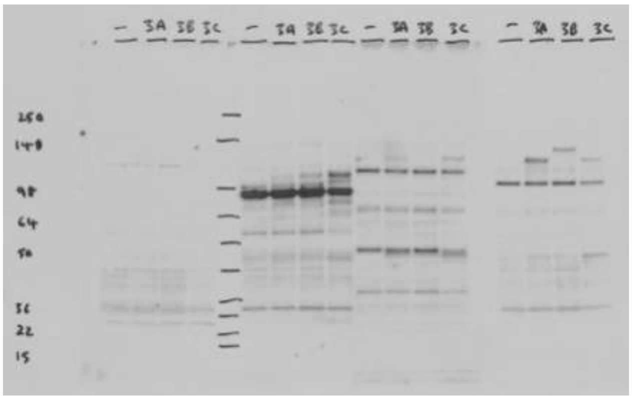

(Left to right) HC2 – HC4 – HC12 – HC25

**Blot 19**

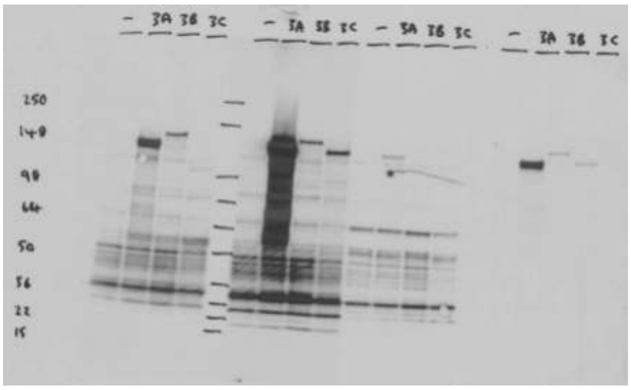

(Left to right) MS3 – MS6 – MS14 – MS28

**Blot 20**

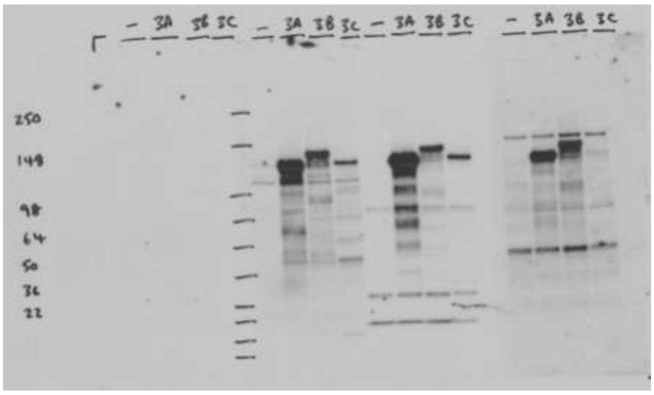


(Left to right) HC7 – HC16 – HC18 – HC19

**Blot 21**

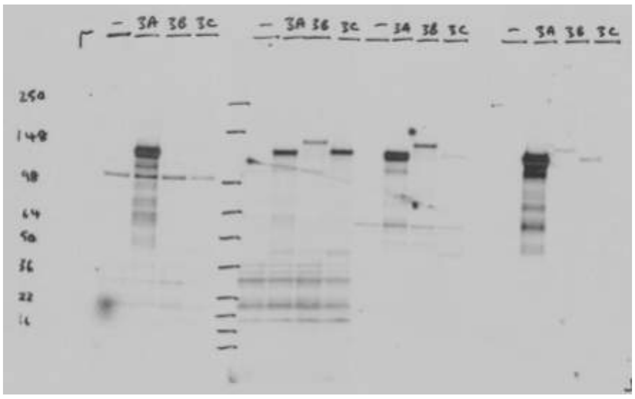


(Left to right) MS2 – MS4 – MS29 – MS30

**Blot 22**

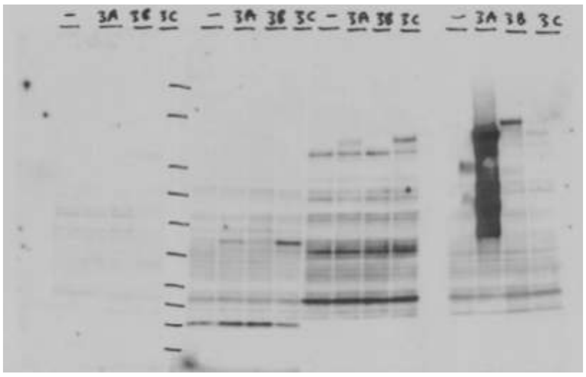

(Left to right) HC14 – HC13 – HC9 – MS5

**Blot 23**

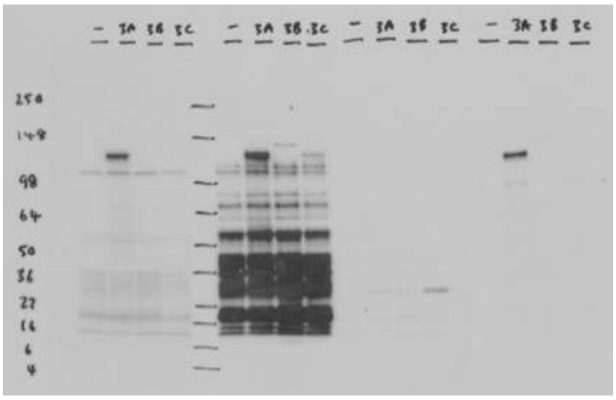


(Left to right) MS1 – MS20 – MS33 – MS34
