# Supplementary material for: Heightened Epstein-Barr virus immunity and potential cross-reactivities in multiple sclerosis
Source: PLoS Pathog. 2024 Jun 6;20(6):e1012177. doi: 10.1371/journal.ppat.1012177 (PMC11156336; doi:10.1371/journal.ppat.1012177)
Supplement: Attachment — Submitted filename: 250324 Response_to_reviewers.docx [file ppat.1012177.s014.docx]

We thank the reviewers for their careful analysis of our work, your comments have enabled us to greatly improve the manuscript. Please find below our answers to your queries and details of changes to the revised manuscript.

**Reviewer #1:** Points to address

*- It would be important to add information about the disease duration in pwMS: since pwS in this study were all untreated, it is likely that most of them were in the early phases of the disease, an information of some relevance for disease pathophysiology..*

Response: We agree with the reviewers and thank them for this suggestion. We have added additional clinical data on disease duration and EDSS score in the manuscript (new Supplementary Table 1) and now describe these parameters in the manuscript (lines 184 to 188) and their calculation in the methods (lines 590-592)

*- Lack of ANO2 (Tengvall, PNAS 2019) and GLIALCAM (Lanz, Nature 2022) and other proteins in the candidate CNS autoantigens pool (ma viene parzialmetne ammesso nella discussion L581-588)*

Response: Our experiments were planned a few years ago, at which time we had to finalise the panel of CNS targets because the MVA viruses had to be constructed. This took place before the ANO2 and GlialCAM papers were published. Although we did not test these two proteins in our work, we tested nine other CNS proteins and showed several are cross-reactive with EBV-specific T-cells. Interestingly, others are not. These results will help to direct future studies towards the most relevant targets and show cross-reactivity between EBV and the CNS is a much broader phenomenon than previously appreciated. MOG, which we show for the first time is cross-reactive with EBV, appears to be a particularly interesting target given that most people have cross-reactive responses to this protein.

In response to the reviewer’s comment, we have added text to the discussion (lines 533-536) about the recently discovered proteins and we highlight that although we did not test these particular proteins, our data clearly shows that cross-reactivity extends beyond them and involves many other CNS proteins. Our results are novel and highly relevant for EBV and MS researchers.

*- L 369-70 How do the authors explain the enhanced recognition of CNS antigens in HD vs
MS? (anche questo parzialmente discusso L596-605)*

Response: The presence of CNS autoantigen-specific antibodies and T cells has also been detected by others in the blood from healthy individuals (e.g. Bielekova *et al.* 2004 J Immunol, Tengvall et al. 2019, Thomas et al. 2023). The presence of such responses is therefore, by themselves, insufficient to cause disease. We hypothesise that a second “hit” is needed to trigger disease. For example, a blood-brain barrier permeabilisation event that allowed these cells to enter the CNS in large numbers might be required.

It may therefore be the case that in pwMS a substantial number of cross-reactive T-cells are localised to tissues, including the CNS, explaining their lower frequency in the blood. We propose this as a possible explanation in the discussion (lines 563-567).

“It is also important to consider that these cells were detectable in the blood and, whilst the extent of CNS cross-reactivity in the EBV-specific T cell repertoire is large in both groups, the differences between HC and MS individuals may instead lie with the ability of these cells to migrate across the blood-brain barrier or other factors such as regulation of their activity by peripheral tolerance mechanisms.”

**Reviewer #2:** Specific Comments

*Fig. 1. Would be valuable to compare EBV loads during active flares of MS, rather than during stable periods.*

Response: We agree with the reviewer, however in reality this is very difficult to achieve due to delays between symptoms occurring, attendance of patients at neurology clinics and subsequent sampling. Reliable MRI measures obtained close to date of sampling were unfortunately not available for the individuals in our study. Instead, we have added extra clinical data to the manuscript on EDSS and disease duration which are reliable measures of disease stage and severity at sampling. These measures are reported in the revised manuscript in the new Supplementary Table 1. Correlation with EBV load and antibody responses are also shown in a new Supplementary Figure 1. Additional text has also been added to the results and methods sections (lines 184-188 and 590-592)

*Fig 2A. It would be helpful to show the full Western blots, including the low molecular wt fractions, to understand the immunogenicity of LCLs in these patient cohorts.*

Response: We thank the reviewers for this suggestion and include the full Western blots for transparency with the donor initials redacted for confidentiality. We state in the manuscript that they are available as supplementary appendices (lines 650-651). They are provided as “S1_Appendix.pdf” and “S2_Appendix 2.pdf”.

*Fig. 2C should show individual data points in bar graphs.*

Response: Fig 2C shows the percentage of the donors in each category (HC, MS, POST-IM) with or without a band corresponding to the latent antigen-specific antibodies. It is therefore a summary statistic and so individual data points cannot be shown.

*Fig. 3E should have p-value calculation for the CD8 INFgamma. Also, legend has asterisk for p-values, but there are no asterisks in the Figure.*

Response: We thank the reviewer for this comment. The results in panels 3C-3F were analysed using the Kruskal-Wallis test with Dunn’s post-hoc correction but only significant comparisons were shown for clarity. We have amended the legend to make this clear

“Statistical significance for the data shown in panels C-F was calculated using the Kruskall-Wallis test with Dunn’s post-hoc test for multiple comparisons; only significant p values are shown.”

*Fig 4. It is not clear that there is any specific preference for neuronal antigens, and that any antigen expressed in this manner would raise a strong T-cell response. A better negative control would be to express a non-neuronal antigen in the MVA infected B-cell blasts.*

Response: The lack of T-cell response against some of the CNS antigens we expressed using recombinant MVA viruses (MBPv8, CNP, Claudin, CRYAB) clearly shows that not every antigen expressed in our system is capable of raising a strong T-cell response. They effectively act as the negative controls the reviewer suggests, providing reassurance that the T-cell responses that we do detect (e.g. MBP, MOG, PLP, TAL-H, MOBP and EBV-EBNA1) are antigen specific.

**Reviewer #3:**

*1. The titer of EBNA2 and 3s antibodies would be useful to generate beyond a simple yes/no from the westerns. This would add more to the literature around the levels of anti-EBNA (1, 2, 3s) antibodies that correlate with disease.*

Response: We agree that it would have been useful to quantitatively measure EBNA2 and EBNA3 antibody titres. However, commercial ELISA assays capable of doing this do not exist. We have attempted to generate our own, by over-expressing and purifying these proteins in pro- and eukaryotic systems, but have never succeeded. EBNA2 contains a 40aa poly-proline repeat (see genbank YP_401644) and the EBNA3 proteins migrate 50% larger than their predicted size on reducing SDS-PAGE indicating substantial charge and/or secondary structure.

We therefore developed recombinant Modified Vaccina Ankara viruses to express these antigens in B cell lines, which is the closest physiological expression of these antigens we could achieve. The technical difficulty of expressing EBNA2 and EBNA3 is why relatively few studies have investigated antibody responses to EBV antigens other than EBNA1. Our work therefore provides a way to tackle this problem. To address the valid point made by the reviewer, we have added text to the discussion (lines 450-455).

“Whilst the “EBNA print” method used for analysis of EBNA2 and EBNA3-specific antibody is only semi-quantitative, it nonetheless provides a broad and visually clear idea of the frequency of antibody responses to a wider array of the EBV latent proteins. It is an extremely useful tool given the difficulty in producing these antigens recombinantly and the lack of commercially available serological testing kits for EBNA2 and EBNA3-specific IgG.”

*The experiments analyzing cross-reactivity of T cells between EBV LCLs and auto-antigens are interesting, but lack mechanistic depth - perhaps the experiments that indicate that the order of EBV LCL or auto-antigen used to generate the polyclonal responses should be moved from supplement to main tables and figures - these are important/interesting pieces of this puzzle despite not having clear explanations for why these differences exist.*

Response: We thank the reviewer for this helpful suggestion. We have moved Supplementary Table 3 in the original manuscript to the main text and this is now Table 3 in the revised manuscript.

**Minor Issues**

**Reviewer #1:**

*- L 159-160 better “no difference in peripheral EBV reactivation (lytic phase) between
inactive MS and HD”*

Response: We thank the reviewer for this comment, please find this updated at lines 184-185 in the revised manuscript.

*- L 377 typo “focussing”*

Response: We have altered this to the American English spelling of the word here and throughout the manuscript (line 358).

*- L 439 typo “in infection”*

Response: We thank the reviewers for noticing this mistake.

**Reviewer #2:**

*Some of the supplemental figures may be more impactful in the main body of the manuscript.*

**Reviewer #3:**

*As noted above, Supp Table 2 and other Supp Figs should be included in the main part of the paper.*

Response: We thank the reviewer for this comment and have now moved Supplementary Table 2 and Supplementary Table 3 into the main text. These are now Table 2 and Table 3 in the revised manuscript.

**Administrative points for the journal**

1. Figures now uploaded as separate TIFF files

2. Minimal dataset now provided as supporting information.

3. Author summary has been prepared between abstract and introduction..

4 Copyright and trademark symbols have been removed from the text.

5. Supporting information legends added to main text.

6. The full name of the research ethics committee has been provided in the manuscript.

7. Images within Figure 4B were prepared by hand by the first author and are not copyrighted.
